# Upper limb rehabilitation system based on virtual reality for breast cancer patients: Development and usability study

**DOI:** 10.1371/journal.pone.0261220

**Published:** 2021-12-15

**Authors:** Zijun Zhou, Jiaxin Li, He Wang, Ze Luan, Yuan Li, Xin Peng

**Affiliations:** 1 Breast Surgery, Jilin Province Tumor Hospital, Jilin, China; 2 School of Nursing, Jilin University, Jilin, China; University of Catanzaro: Universita degli Studi Magna Graecia di Catanzaro, ITALY

## Abstract

**Background:**

Functional exercise is crucial for breast cancer patients after surgery, and the use of virtual reality technology to assist patients with postoperative upper limb functional rehabilitation has gradually attracted the attention of researchers. However, the usability of the developed rehabilitation system is still unknown to a large extent. The purpose of this study was to develop a virtual reality upper limb rehabilitation system for patients after breast cancer surgery and to explore its usability.

**Methods:**

We built a multidisciplinary team based on virtual reality and human-computer interaction technology and designed and developed an upper limb function rehabilitation system for breast cancer patients after surgery. Breast cancer patients were recruited from a grade III-a general hospital in Changchun city for the experiment. We used the System Usability Scale to evaluate the system availability, the Presence Questionnaire scale to measure the immersive virtual reality scene, and the Simulator Sickness Questionnaire subjective measurement scale for simulator sickness symptoms.

**Results:**

This upper limb rehabilitation system hardware consisted of Head-mounted Display, a control handle and notebook computers. The software consisted of rehabilitation exercises and game modules. A total of 15 patients were tested on this system, all of whom were female. The mean age was 54.73±7.78 years, and no patients were excluded from the experiment because of adverse reactions such as dizziness and vomiting. The System Usability Scale score was 90.50±5.69, the Presence Questionnaire score was 113.40±9.58, the Simulator Sickness Questionnaire-nausea score was 0.93±1.16, the Simulator Sickness Questionnaire-oculomotor score was 0.80±1.27, the Simulator Sickness Questionnaire-disorientation score was 0.80±1.27, and the Simulator Sickness Questionnaire total score was 2.53±3.40.

**Conclusions:**

This study fills in the blanks regarding the upper limb rehabilitation of breast cancer patients based on virtual reality technology system usability research. As the starting point of research in the future, we will improve the system’s function and design strictly randomized controlled trials, using larger samples in the promotion, to evaluate its application in breast cancer patients with upper limbs and other physiological functions and the feasibility and effects of rehabilitation.

## Introduction

Breast cancer is the most common malignancy threatening women’s health. The latest report of the International Agency for Research on Cancer shows that in 2018, the number of new cases of breast cancer increased by approximately 2.09 million worldwide, accounting for 24.2% of all cases of female cancer [1], while the number of new cases in China accounted for approximately 37% of all cases of female cancer [2].

Surgery is the main treatment for breast cancer [3–5]. Patients will have different degrees of symptoms, such as swelling, pain, numbness, and raising difficulty, post-traumatic stress disorder, axillary web syndrome, cancer-related fatigue, become long-term health problems for patients [6–16]. Postoperative upper limb functional exercise can gradually replace the role of armpit tissue by enhancing muscle strength, fully activating deltoid muscle, deep scapular muscle and latissimus dorsi muscle, etc., and can effectively reduce the incidence of postoperative complications such as subcutaneous blood and fluid accumulation in the affected limb, necrosis of the skin flap and severe upper limb edema, which can prevent the functional recovery of the operative side limb [17, 18]. Rehabilitation exercise can improve muscle pump, promote lymphatic fluid flow, aerobic training can increase intra-abdominal pressure, to promote chest duct pump blood, improve lymphatic reflux [19]. It is safe and effective for patients at risk of breast cancer–related lymphedema and those with BCRL at all points in the life trajectory [14, 20, 21]. In addition, rehabilitation exercise can stimulate the release of β -endorphin, exciting the patient’s central nervous system, relieve their pain while improving sleep and mood, also can make nervous system release inhibition muscle tension and mental depression of the micro electrical stimulation, in order to eliminate or reduce fatigue [22, 23], improve exercise compliance [24], its role in reducing breast cancer-related fatigue has been demonstrated [25, 26]. In addition, after upper limb exercise and muscle relaxation training, the affected shoulder function and health-related quality of life were significantly improved, and early rehabilitation treatment (including range of motion of shoulder and intensive exercise) could improve the range of motion of shoulder after breast cancer surgery [26, 27]. Therefore, it is very important to develop a scientific functional exercise plan for breast cancer patients after surgery and urge them to carry out gradual functional exercise for the recovery of upper limb and shoulder joint function and reduce the incidence of complications.

VR technology refers to the use of computer systems and sensor technology to generate a three-dimensional environment and to create a new pathway of man-machine communication by mobilizing users’ various senses (vision, hearing, touch, smell, etc.) to enjoy a more real, immersive feeling. VR is characterized by immersion, imagination and interaction [28]. It has been a breakthrough in the field of artificial intelligence; medical care, rehabilitation, nursing and other fields have been spreading [29–31], especially in the prevention and control of the COVID-19 normalized background. Through development platforms based on VR home rehabilitation of breast cancer patients with postoperative management, hospital nursing care and rehabilitation management continue to help patients recover with their families because home rehabilitation management is of great significance.

At present, VR technology has been carried out in the field of rehabilitation of breast cancer patients. With the development and diversification of technology, an increasing number of studies are combining it with robots, 3D motion-sensing cameras, machine learning and other technologies to provide process data feedback on the rehabilitation time limit and connotation quality to make the rehabilitation process more accurate and achieve remarkable effects.

Usability evaluation is an indispensable link in the process of medical product development and one of the key factors for the successful implementation of telemedicine [32]. The International Standardization Organization 9241–11 international standard defines usability as the effectiveness, efficiency and user subjective satisfaction of a product when it is used for a specific purpose by a specific user in a specific use environment [33]. Usability expert Jakob Nielsen believes that usability testing is carried out at all stages of the product design and development process, including early paper prototypes, rapid prototyping, and postproduction [34]. Usability evaluation can bring great value for the patient, increase productivity, enhance user well-being, avoid pressure, increase accessibility and reduce injury risk; therefore, scholars have suggested that before large clinical trials, it is necessary to invest a certain amount of time and resources in usability evaluation. Due to the three characteristics of VR: Immersion, Imagination and Interaction, the connotation of product usability developed based on this technology and its derivative technology has been supplemented and updated. Presence is a subjective feeling rather than an objective reality, which is exactly what the VR system aims to achieve so that the "virtual" can achieve a "real" effect. Cybersickness is one of the most common side effects in VR applications, Statistics show that more than 60% of users will experience screen sickness when using VR devices, which will bring an unpleasant experience to users and reduce the time and frequency of using VR devices [35]. The degree of presence and the severity of cybersickness was gradually covered in usability study. To the best of our knowledge, at present, there is still no research on the usability of upper limb rehabilitation for breast cancer based on VR.

This study aims at VR technology and computer graphics technology, human-computer interaction technology, simulation technology, multimedia technology, sensor technology, network technology to develop a system for exercising upper limb function for breast cancer patients and performs usability study to provide a basis for iterative development and maintenance in the future. In this way, the efficiency and quality of the whole rehabilitation system can be improved, and an effective reference can be provided for data-driven intelligent home rehabilitation.

## Methods

### Formation of a multidisciplinary team

Ten people were involved in a multidisciplinary team, including a breast surgeon (1 person), rehabilitation therapists (2 people), nurses (2 people), postgraduates (3 people) and technical engineers (2 people), on the basis of consulting and summarizing the contents of the guidelines, in accordance with scientific, innovative, practical and feasible principles. Combined with the results of previous research and literature research, the content of the upper limb rehabilitation system was preliminarily conceived, and the functional details were further determined through discussion and brainstorming.

### System development

Hardware side: The hardware of the system is an HTC VIVE Pro2.0 device (including 2 handle controllers, 2 infrared base stations and 1 HMDs device), and the helmet is connected to a computer (Dell Ravener G15 laptop, 3060 graphics card, Intel Core i7 processor, Windows 10 operating system). The handle controller and HMDs use Csharp programming language for secondary development, based on an 850-nm infrared optical orientation method, through the base station to capture the patient’s head and control the handle position and to track the coordinates of the upper limb activity information. The upper limb activity trajectory was calculated and compared with the preset movements of rehabilitation training in the movement database to identify the movements and send the data to the cloud server in real time.

Software side: The virtual simulation was developed with the Unity3D game engine and written in C # script language. Component design is completely independent of the hardware and operating system development environment, and the application platform module is relatively independent and convenient for secondary development.

We put the code for the experiment on the github open source site for easy access: https://github.com/2202jasmine1024/breast-cancer

### System function design

A VR-based postoperative rehabilitation system for breast cancer was preliminarily constructed and was mainly presented in the form of web pages and C/S structures, including rehabilitation exercise modules and puzzle game modules.

In the rehabilitation exercise module, the rehabilitation exercises were set by the hospital rehabilitation therapists and nurses; they covered making a fist, screw lifting of the wrist, elbow flexion and around the shoulders, touching the ear, climbing a wall, back-handing, and outreach, comprising 10 rehabilitation actions according to the situation of patients with different clinical multidisciplinary teams set in two different mode of rehabilitation (for details, see Table 1). Each section must be completed in eight beats, and after 32 reps, the program will automatically jump to the next section. During the completion of each session, when the progress reaches 50% and 100%, there will be an encouraging voice prompt, such as: Come on, victory is in sight! Very good, you have completed the action of this section! Congratulations you have completed all the training!

**Table 1:** Exercise model.

	Exercise purpose	Appropriate people	Rehabilitation exercise
Mode 1	1. Move fingers and wrists to relieve stiffness and numbness; 2. Contraction of the upper extremity is carried out, and the effect of muscle pump is used to promote the subsidence of swelling and lymphatic reflux of the affected limb.	Patients who have not yet pulled out their drainage tube or still have fluid and gas accumulation in the skin flap shortly after surgery.	Three sections: (1). Fist clenching, (2). Wrist twisting, (3). Elbow bending.
Mode 2	1. Promote the subsidence of swelling and lymphatic reflux of the affected limbs; 2. Enhance the muscle strength of the affected side; 3. Strengthen shoulder joint activity, release and prevent adhesion, and maximize the recovery of the range of motion of the shoulder joint.	Patients with their postoperative drainage tube pulled out and no fluid and gas accumulation in the skin flap.	Ten sections: (1). Fist clenching, (2). Wwrist twisting, (3). Eelbow bending, (4). Lifting, (5). Shoulder circling, (6). Ear touching, (7). Wall climbing, (8). Back handing, (9). Head holding, (10). Abduction.

**Fig 1:**
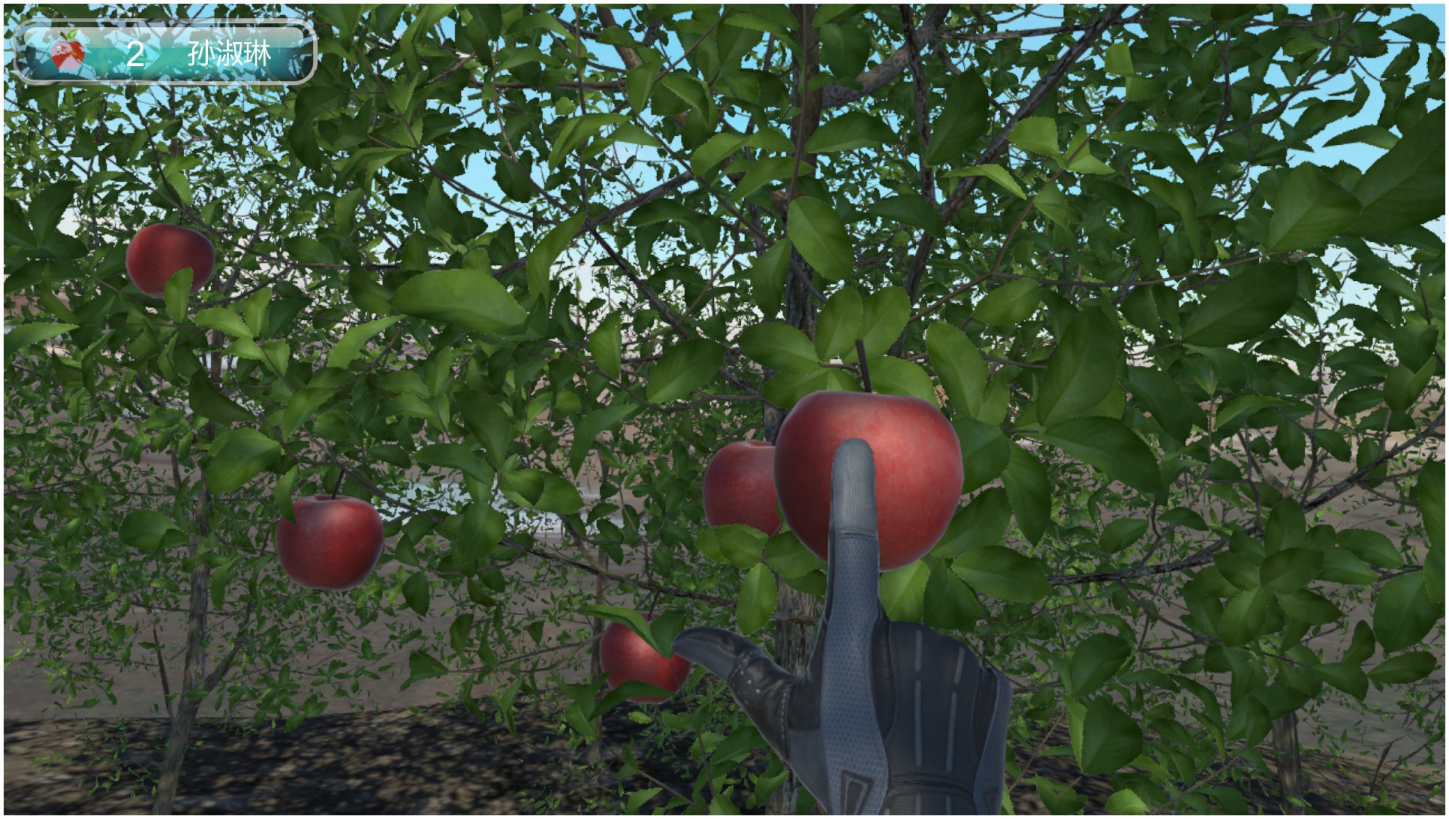
Screenshot of virtual reality rehabilitation interface (1).

**Fig 2:**
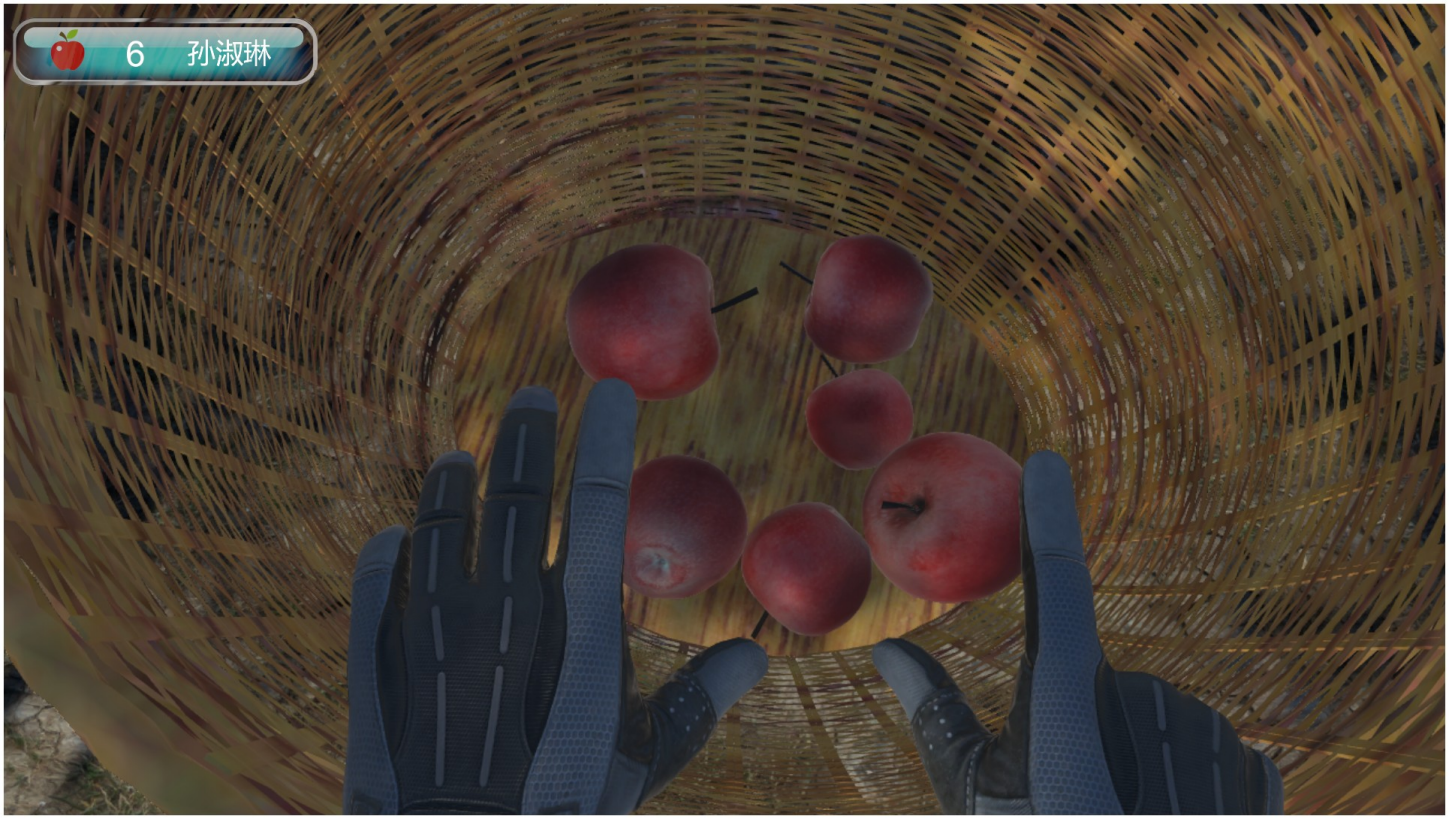
Screenshot of virtual reality rehabilitation interface (2).

In addition to the rehabilitation exercise scene, we embedded a puzzle game, which is set in an orchard and plays soothing music in the ear. Figs 1 and 2 show a user’s view of the hands of their virtual characters, which are controlled by their movements. Patients complete the game after picking 32 apples.

### Usability study

#### Subjects and sampling method

From June 12, 2021, to June 27, 2021, breast cancer patients in the Breast Surgery Department of a Grade A hospital in Changchun City, Jilin Province were randomly sampled. The inclusion and exclusion criteria are shown in Table 2.

**Table 2:** Inclusion and exclusion criteria.

Inclusion criteria	Exclusion criteria
(1) At least 18 years old;	(1) Complicated with serious diseases of the heart, lung, kidney, liver and other organs;
(2) Diagnosed with breast cancer on the basis of pathological examination;	(2) Patients with previous symptoms of dizziness, vestibular disease or cybersickness;
(3) Radical mastectomy, modified radical mastectomy, breast-conserving surgery, axillary lymph node dissection and simple mastectomy;	(3) Central and peripheral nerve injury;
(4) The pathological stage of the tumor was I-III;	(4) Patients with eye disease, such as cataract, or glaucoma;
(5) Able to carry on upper limb and whole-body physical activity	(5) Patients with serious mental or psychological diseases.
(6) Able to use smart devices such as mobile phones and computers;	
(7) Informed consent and voluntary participation in this study.	

#### Sample size calculation

According to the number of participants and the problems identified in the percentage relationship curve [36], 10 patients were able to identify 80% of usability problems, and 15 participants were able to identify 100% of usability problems; therefore, this study’s sample size was in the range of 10 to 15 people.

#### Permissions and ethics approval

All patients signed an informed consent form. This study was approved by the Ethics Committee of the Nursing College of Jilin University, registration number: 2020091805.

#### Experimental design

**Fig 3:**
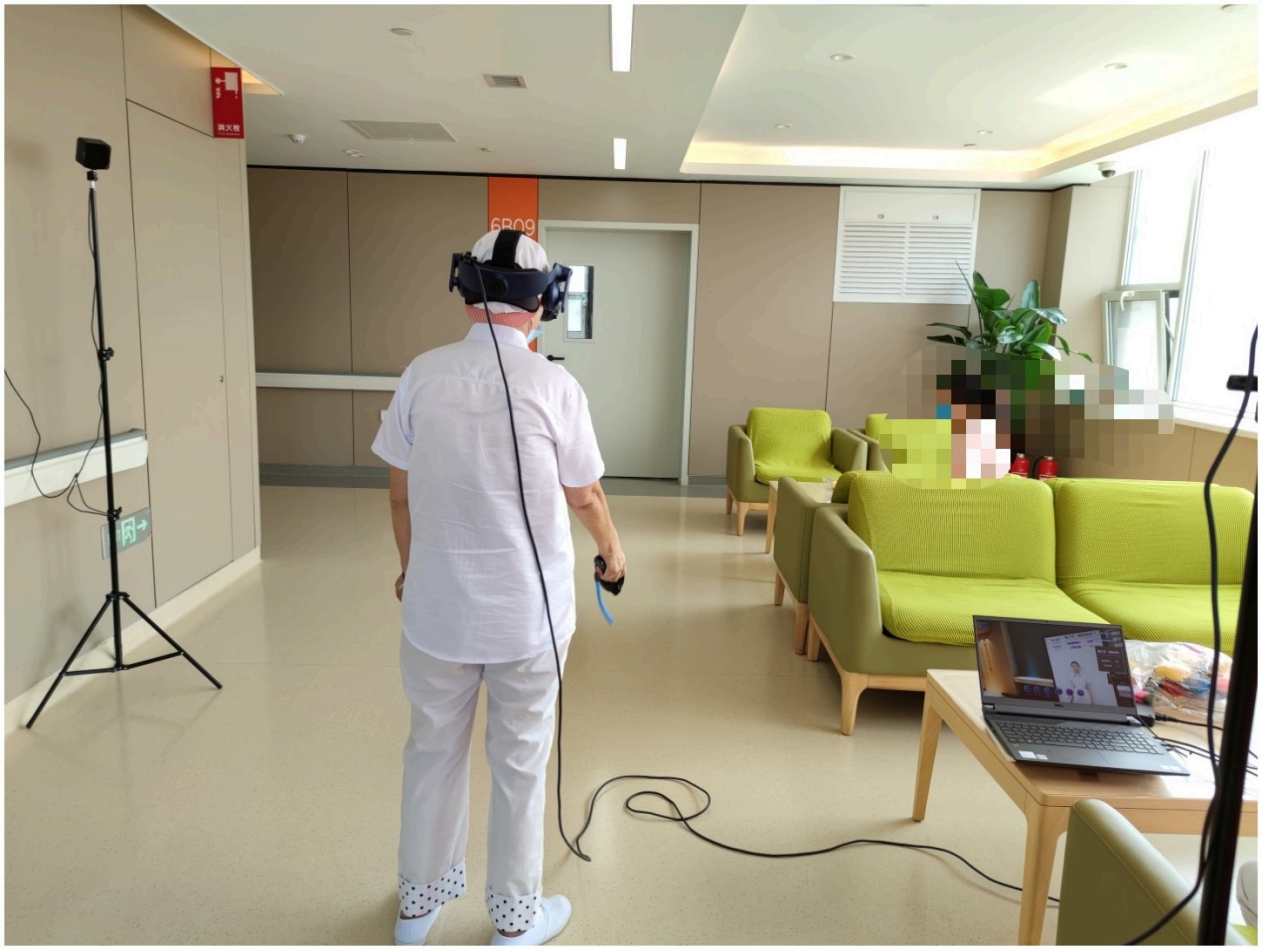
Picture of a patient using the rehabilitation system in the ward.

In a room with an area size of 5 m * 5 m, the room temperature was kept at 22~24 °C and weak light. Each participant was asked to fast for two hours prior to the experiment, which was designed to distinguish between postprandial exercise and cybersickness. A doctor (Zijun Zhou) and a nurse (He Wang) from the multidisciplinary team introduced the purpose of the study, the inherent characteristics of the system, module functions and matters needing attention to the participants in the ward, the two researchers demonstrated the login and use methods and informed the participants of the tasks to be completed later. Subjects performed a basic warm-up 10 minutes before the experiment to prevent injuries during the exercise. After the experience, participants rested in the ward for 15 minutes while filling out a questionnaire provided by another researcher. Two postgraduate students (Jiaxin Li and Ze Luan) from the multidisciplinary team, guided the participants in filling out the scale, and conducted unified training for postgraduate students before the survey to make them understand the research objectives and methods During the investigation, two postgraduate students issued questionnaires and guided patients to fill in the scale by themselves with unified guidance language. If there was any doubt about the content of the scale, they explained it in consistent language. Researchers paid close attention to the whole process of the experiment to ensure the patients’ personal safety. Fig 3 shows a patient using the rehabilitation system in the ward.

#### Outcome indicators

We used a general information questionnaire to collect the patients’ age, nationality, marital status, educational level, pathological staging, pathological type, treatment and other information.

The SUS is used to evaluate the usability of the system [37], which is a validated measurement method of learnability and user satisfaction and is used to subjectively evaluate the usability of interface technology. This survey has excellent reliability (0.85). Each item is scored on a 5-point Likert scale, ranging from 1 (strongly disagree) to 5 (strongly agree). For odd items, the score is reduced by 1, and for even items, the score is reduced by 5. Therefore, the score for each item ranges from 0 to 4, the score is summed and multiplied by 2.5, and the final score ranges from 0 to 100.

The SSQ was used to evaluate users’ symptoms of cybersickness [38]. The SSQ has been shown to be reliable in healthy adults (0.80). It contains 16 small symptoms, including nausea, oculomotor nerve discomfort and disorientation as 3 significant symptoms, with all events scored on a 4-point Likert scale: 0 (no symptoms), 1 (minor), 2 (medium), and 3 (severe), with scores multiplied by the corresponding weight. To evaluate the three major symptoms, the higher the total score, the higher the degree of motion sickness.

The PQ was used to evaluate users’ sense of presence and immersion [39], which emphasized the characteristics of involvement and immersion in the simulation environment. This survey has excellent reliability (0.88) and included 17 items. Each item was scored on a 7-point Likert scale, ranging from 1 to 7. The higher the score, the better the user’s sense of presence and presence.

### Statistical method

Microsoft Excel 2019 and SPSS 24.0 statistical software were used for data analysis. Descriptive statistical methods were used in this study was descriptive analysis: frequency and constituent ratio were used to describe the general information treatment status and other data. Median and quartile spacing were used to describe the SUS score, SSQ score, PQ score.

## Results

A total of 15 patients were recruited in this study, all female, with an average age of 54.73±7.78 years. See Table 3 for the general demographic information.

**Table 3:** General demographic information (N = 15).

Characteristic	Groups	N(%)
**Age (mean±SD)**	54.73±7.78.	
**Nationality**	Han nationality	14(93.3)
	Minority	1(6.7)
**Marital status**	Married	14(93.3)
	Divorce	1(6.7)
**Educational level**	Primary school	4(26.6)
	Junior high school	2(13.3)
	Senior high school	6(40.0)
	Bachelor or above	3(20.0)
**Residence**	Urban	11(73.3)
	Rural	3(20.0)
	Town	1(6.7)
**Occupation**	Farmer	3(20.0)
	Workman	1(6.7)
	Employees of enterprises and public institutions	1(6.7)
	Freelance	3(20.0)
	Unemployed	1(6.7)
	Retired	6(40.0)
**Monthly income**	<¥1000	4(26.6)
	¥1000~2999	6(40.0)
	¥3000~4999	1(6.7)
	¥5000~9999	3(20.0)
	≥¥10000	1(6.7)
**Payment types**	Self pay	1(6.7)
	Medical insurance	9(60.0)
	The new rural cooperative medical insurance	5(33.3)
**Pathological stage**	Stage I	3(20.0)
	Stage II	10(66.7)
	Stage III	2(13.3)
**Pathological type**	Noninfiltrative carcinoma	2(13.3)
	Invasive nonspecific carcinoma	11(73.3)
	Invasive specific carcinoma	2(13.3)
**Treatment method**	Surgery	15(100.0)
	Chemotherapy	14(93.3)
	Radiotherapy	8(53.3)
	Endocrine therapy	2(13.3)
**Modus operandi**	Modified radical mastectomy	11(73.3)
	Breast-conserving surgery	4(26.6)
**Type of smart device previously used**	Smartphone	15(100.0)
	Tablet computer	1(6.7)
	Desktop computer	1(6.7)

Abbreviation: SD = Standard Deviation.

As shown in Table 4, the SUS score was 90.50±5.69, the PQ score was 113.40±9.58, the SSQ-N score was 0.93±1.16, the SSQ-O score was 0.80±1.27, the SSQ-D score was 0.80±1.27, the PQ score was 113.40±9.58, and the SSQ total score was (2.53±3.40).

**Table 4:** SUS, PQ, and SSQ scores.

Patient ID	SUS	PQ	SSQ-N	SSQ-O	SSQ-D	SSQ-TS
**1**	90.00	99.00	0	0	1	1
**2**	85.00	116.00	0	0	0	0
**3**	90.00	119.00	0	0	0	0
**4**	80.00	119.00	0	0	0	0
**5**	95.00	90.00	1	1	1	3
**6**	100.00	119.00	3	4	2	9
**7**	82.50	119.00	1	0	0	1
**8**	90.00	119.00	0	0	0	0
**9**	92.50	117.00	1	1	0	2
**10**	97.50	119.00	0	0	0	0
**11**	97.50	99.00	1	0	0	1
**12**	85.00	119.00	3	1	4	8
**13**	90.00	119.00	3	3	3	9
**14**	90.00	109.00	0	0	0	0
**15**	92.50	119.00	1	2	1	4
**Mean±SD**	90.50±5.69	113.40±9.58	0.93±1.16	0.80±1.27	0.80±1.27	2.53±3.40

Abbreviation: SUS = System Usability Scale; PQ = Presence Questionnaire; SSQ = Simulator Sickness Questionnaire; HMD = Head-mounted Display; SD = Standard Deviation; N = nausea; O = oculomotor; D = disorientation; TS = total score.

During the trial, no adverse events were reported, and no patients withdrew for any reason.

## Discussion

Using VR technology arm rehabilitation exercise is a new kind of method for the postoperative rehabilitation of breast cancer. When experiencing a new environment, novelty, immediacy and uniqueness can make people more excited. People can give more attention to completing a task or experience using VR devices through multisensory interactive approaches such as seeing, hearing [40, 41]. Patients can be motivated to participate by adding fun and gamification, in addition, game design elements and game principles can be added to the task to increase patient exercise compliance. According to the characteristics of movement disorders of patients, a flexible and personalized rehabilitation design makes it possible to provide step-by-step rehabilitation treatment. Low-cost virtual rehabilitation systems can be used as adjuvants to regular rehabilitation with less direct supervision by doctors or can be considered remote or home rehabilitation tools. The use of motion sensors in conjunction with a VR system for rehabilitation allows the functional assessment and digital tracking of patients, which adds beneficial elements to current rehabilitation strategies. To our knowledge, this is the first study to explore the development and availability of a breast cancer rehabilitation exercise system, which has not been previously reported.

There are many rehabilitation systems based on VR and its derivative technologies to assist breast cancer patients to recover their upper limb function, quality of life and psychological state. Such as House et al. [42] developed a virtual robot rehabilitation system platform, Brightarm Duo, which provides patients with multisensory stimulation through nine sports games and puzzle games for passive shoulder movement, motion control, single-hand gripping strength training, and emotional and cognitive training. After intervention, the patients showed improvements in upper limb strength, upper limb function, pain level, emotion management and cognitive function. Feyzio G Lu et al. [43] used an Xbox 360 Kinect, a 3D motion camera matched with a VR system, to move patients off of the handgrip during training and to implement task-based sports programs combined with shoulder passive motion. There were significant decreases in pain level and fear of exercise in the test group. The upper limb function score and shoulder joint range of motion increased. Axenie et al. [44] combined VR and machine learning technology to develop a sensorimotor function rehabilitation system for multiple neuropathies caused by chemotherapy. Virtual characters on the screen increased the patient’s sense of existence and identity and combined the accurate mapping of motor characteristics and multimodal perception with the consistency and effectiveness of internal model prediction. Park et al. [45] developed a digital healthcare system based on augmented reality technology and motion sensor technology, through tracking and positioning 25 joints of upper and lower limbs, using Xbox One Kinect for Windows records and feedback the number of repetitions and precision of exercise tasks in real time. Doctors can remotely prescribe exercise prescriptions to users by referring to uploaded data, which can be modified and calibrated according to patients’ recovery and exercise effects. Participants can carry out daily exercise according to doctors’ prescriptions. But the technology used in this study mainly focused on the positioning of joint angles Tracking the trajectory, rather than placing the patient in an immersive world, remains fundamentally different from our focus.

In addition, VR and its derivative technologies have been widely used in the diagnosis of complications [13], palliative care [46, 47], pain relief [48], reduction of chemotherapy side effects [49], psychological intervention [41, 50]. Such as A. de Sire et al. [14] and Invernizzi et al. [13] used an augmented reality tool 3D laser Scanner, which uses a triangulation mechanism to project a laser point onto the upper limb to be measured. The sensor measures the distance to the surface of the object to detect THE PATIENT’s BRCL, achieving an accurate, repeatable, reliable and economical diagnosis. Helene Buche et al. [51] confirmed that no matter immersive or participatory VR, it is different from traditional emotion regulation tools (such as music, Yoga), can be provided to the patients engaged, through visual will be isolated from the medical environment, patients with change the users’ perception of time, a distraction from the tense unpleasant stimulus, are titer induced mood, improve the well-being, reduce anxiety, it allows the individual’s attention on the virtual experience, scattered from the tense environment unpleasant stimulus.

SUS was originally designed to evaluate the usability of products and services, including hardware, software, mobile devices, websites, and applications. Due to the combination of certain elements of the rehabilitation system developed in this study, and the increasing number of studies using SUS to test medical devices and products [32, 52, 53], we chose SUS to facilitate comparisons with similar and different products or devices. An SUS score above 70 is considered acceptable usability, and a score above 85 is considered excellent usability. A score above 90 is considered truly excellent usability, while a score below 50 indicates an unacceptable usability [37]. The SUS study scored an average of 90.50±5.69, although it is still in its early development stage, but compared with other systems, the SUS system scored higher than the average [54], indicating that the system of usability, ease of use and learnability and confidence was acceptable, but there was still room for improvement. Possibly due to the higher number of women diagnosed with breast cancer in the region Women are often less likely to use Internet electronic products in the age range of 40–70 years because their cultural level is low, their learning ability is weak, and they have conservative and cautious attitudes when accepting new things; therefore, the next step in the process of improvement should consider the characteristics of the audience and the patients’ attitudes and preferences as the basis of iterative improvement.

The VR system uses hardware devices such as HMDs, Cave Automatic Virtual Environment ^™^ and good software design to build a sense of presence, which allows individuals to focus on the virtual experience. Such participation in immersive tasks is called interactive potential [55], and the strength of presence is also an important indicator to measure the usability of VR systems. The PQ score of this study (113.40±9.58) was close to the full score, which was at a high level. Studies have shown that a high sense of presence and immersive participation can enhance users’ ability to interact with the virtual environment, and the quality of immersion, degree of interaction and personal participation are key parameters that affect system availability [51, 55, 56].

Cybersickness is unavoidable in the use of VR. When visual response processing for user input interaction is delayed, the signals from the eyes, the vestibular system of the inner ear and proprioceptive receptors to the brain conflict with each other, and motion sickness is triggered [57]. Halo screen symptoms may include system characteristics (for example, head tracking, rendering, vision, optical), user interaction factors (lack of motion control, visual acceleration or deceleration, VR experience for a longer duration and frequent head movements in VR games), and personal perception factors (gender, age, history of motion sickness, lack of VR experience) [58]. Therefore, when designing VR scenes and animations, the experience of cybersickness can be reduced according to the influencing factors of cybersickness [59]: (1) the user actively controls his viewpoint and adjusts it; (2) avoid or limit linear or angular acceleration or deceleration to reduce stimulation of the vestibular organs; (3) display visual indicators or movement tracks to users; and (4) dynamically blur the unimportant areas.

The higher the SSQ score is, the more serious the cybersickness is, and a score over 20 indicates that the user has obvious discomfort [38, 60]. Analysis of the total SSQ score shows that the rehabilitation system developed in this study does not cause cybersickness or has a small impact. The SSQ scores were 0.93±1.16, 0.80±1.27 and 0.80±1.27, respectively, among which the nausea score was the highest, which might be related to the patient’s recent chemotherapy, but none of the symptoms were significant. Therefore, in future experiments and applications, the time after chemotherapy should be avoided to distinguish the causes of these symptoms and prevent adverse reactions to chemotherapy from leading to poor use experience of the system. The sweating symptoms of some patients are related to hot weather in summer and the heavy weight of the helmet. Therefore, considering the active exercise undertaken during rehabilitation exercise, in the improvement process, HMDs should be as light, comfortable to wear, stable in position and able to dissipate heat as possible. The device used in this study was an immersive HMDs, which gives the best immersive experience to patients compared with other devices [61] and can cooperate with the handle sensor for action recognition and interaction with virtual objects. Studies have shown that compared to using desktop computers and projectors and other nonimmersive devices, wearing HMDs can cause head pressure, eye fatigue, physical fatigue and halo screen symptoms more easily [62]. Therefore, in this study, some patients showed discomfort of the oculomotor nerve and fatigue, thereby targeting issues for improvement in the future. We will consider using surface TVs instead of HMDs to ensure a sense of immersion and reduce sweating, dizziness, oculomotor nerve discomfort and other symptoms.

In addition, some studies have found that presence scores are inversely correlated with cybersickness symptoms. The more a patient feels in a virtual world, the more likely he or she is to experience motion sickness. This study focuses on usability research, with a sample size that is too small and insufficient data collected to support the exploration of the relationship between SSQ and PQ scores. Therefore, the sample size can be increased in future studies to explore the correlation between SSQ and PQ scores.

## Limitations

First, in terms of participant recruitment, usability study adopts a convenient sampling method with a relatively small sample size, but this is not uncommon in development iterations [63], and participants come from one region, which may lead to selection bias. Second, the patients included in this study were exposed to this VR rehabilitation system for the first time, and the results may have been influenced by novelty effects. Third, only questionnaires were used in this paper to judge the availability of the system and the response to the use of VR equipment, and immediate and objective results could not be recorded. In the future, more comprehensive and scientific measurement methods (such as respiration, heart rate, and electroencephalogram) should be used to quantify the symptoms of cybersickness and immersion in real time. Finally, this paper only conducted a usability study without effect evaluation. In the next step, a strict randomized controlled experiment should be designed to evaluate the application effect of the system platform.

## Conclusion

This study is the first step in the new field of VR-based rehabilitation for breast cancer patients to evaluate the usability and feasibility of the newly developed system, to learn from the results of the study and to provide a reference for future research and development projects in this field. The results show that the VR rehabilitation system is available, feasible and easy to learn for breast cancer patients. Through various human-computer interaction methods, such as sight, hearing and touch, participants adapted to the difficulty level according to the selection of rehabilitation mode based on the state of the affected limb and enjoyed the stimulating, diverse and interesting experience of sports games, which presented both physical and psychological cognitive challenges. The system developed in this study is not applied to commercial promotion; in contrast, it is designed to be used as a starting point in other clinical researchers’ potential, immersive VR and interactive technology to explore the potential for the treatment and rehabilitation of breast cancer postoperative rehabilitation purposes. We look forward to the contributions of other researchers in this field.

## Abbreviations

BRCL: Breast Cancer Related Lymphedema
HMD: Head-mounted Display
PQ: Presence Questionnaire
SD: Standard Deviation
SSQ: Simulator Sickness Questionnaire
SUS: System Usability Scale
VR: Virtual Reality
